# A Systematic Review of Wearable Sensors in Rett Syndrome—What Physiological Markers Are Informative for Monitoring Disease States?

**DOI:** 10.3390/s25216697

**Published:** 2025-11-02

**Authors:** Jatinder Singh, Georgina Wilkins, Athina Manginas, Samiya Chishti, Federico Fiori, Girish D. Sharma, Jay Shetty, Paramala Santosh

**Affiliations:** 1 Department of Child and Adolescent Psychiatry, Institute of Psychiatry, Psychology and Neuroscience, King’s College London, London SE5 8AF, UK; 2 Centre for Interventional Paediatric Psychopharmacology and Rare Diseases (CIPPRD), South London and Maudsley NHS Foundation Trust, London SE5 8AZ, UK; 3 Centre for Interventional Paediatric Psychopharmacology (CIPP) Rett Centre, Institute of Psychiatry, Psychology and Neuroscience, King’s College London, London SE5 8AF, UK; 4 Rush University, Chicago, IL 60612, USA; 5 Department of Paediatric Neurosciences, Royal Hospital for Children and Young People, Edinburgh Bio Quarter, Edinburgh EH16 4TJ, UK

**Keywords:** non-invasive biosensor, wearable sensor, Rett syndrome, multimodal biosensing, sensor integration

## Abstract

Rett syndrome (RTT) presents with a wide range of symptoms spanning various clinical areas. Capturing symptom change as the disorder progresses is challenging. Wearable sensors offer a non-invasive and objective means of monitoring disease states in neurodevelopmental disorders. The goal of this study was to conduct a systematic literature review to critically appraise the literature on the use of wearable sensors in individuals with RTT. The PRISMA criteria were used to search four databases without time restriction and identified 226 records. After removing duplicates, the titles and abstracts of 184 records were screened, 147 were excluded, and 37 were assessed for eligibility. Ten (10) articles remained, and a further two were included after additional searching. In total, 12 articles were included in the final analysis. The sample size ranged from 7 to 47 subjects with an age range of 1 to 41 years. Different wearable biosensor devices were used across studies, with the Empatica E4 wearable device being most frequently used in 33% (4/12) of the studies. All the studies demonstrated a high methodological quality with a low risk of bias. Evidence from wearable sensors, combined with machine learning methods, enabled the prediction of different sleep patterns and clinical severity in RTT. Given the small sample size and the limitations of available data for training machine learning models, we highlight areas for consideration. The review emphasises the need to enhance research on the application of wearable sensors in epilepsy and gastrointestinal manifestations/morbidity in RTT. Increased electrodermal activity (EDA), % of maximum heart rate (HRmax%) and the heart rate to low-frequency power (HR/LF) ratio were identified as physiological measures potentially associated with disease states. Based on the evidence synthesis, the role of physiological parameters and their association with symptom management in RTT is discussed.

## 1. Introduction

Rett Syndrome (RTT; OMIM 312750) is a progressive neurological disorder usually associated with de novo pathogenic variants in the X-linked methyl-CpG-binding protein 2 gene (*MECP2*) [1]. The disorder presents with a myriad of multisystem comorbidities spanning both physical and mental health domains. In the North American cohort, with adequate healthcare, 70% of those with classical RTT survive up to 50 years of age [2]; however, evidence on life expectancy in the UK is limited. Any potential treatment for RTT will likely need to be used in conjunction with adjunct measures that track and monitor disease longitudinally. Clinical outcome assessments (COA) have been used in studies of RTT, and two commonly used measures are the Rett Syndrome Behaviour Questionnaire (RSBQ) [3,4] and the Rett anchored Clinical Global Impression (CGI) scale [5]. Both the RSBQ and the RTT-anchored CGI were used to capture changes with trofinetide compared to placebo [6]. More recently, efforts have been made to ensure that COAs are “fit-for-purpose” for clinical trials in RTT, i.e., can capture clinically meaningful change [7]. However, despite the ubiquity of COAs in studies of RTT [8], due to their subjective nature, COAs in RTT have reliability and consistency issues.

When monitoring specific problems, such as sleep, the use of sleep diaries is prone to observer bias. Evidence also suggests limitations between parental reporting and sleep monitoring in neurodevelopmental disorders such as RTT and Down syndrome [9,10]. Seizures are also common in RTT [11], however there are significant challenges in differentiating epileptic seizures from behavioural changes related to RTT [12] as well as other neurological disorders. Current methods of seizure diaries used as clinical trial outcomes are not ideal. It would therefore be sensible to use other methods, alongside subjective ones, to capture clinically meaningful change, especially measures that can capture data longitudinally. Wearables with accelerometers, gyroscopes, and magnetometers, have been utilised in medical applications [13]. The phenomenon of giant magnetoimpedance (GMI) has led to the development of GMI biosensors that have expanded the field of biomedical diagnostics [14]. These biosensors have paved the way for their use in various medical applications, such as the development of magnetocardiography (MCG) sensors to enhance cardiac diagnostics [15].

### 1.1. Clinical Utility

Non-invasive wearable sensors can monitor symptoms in real-time. In epilepsy, wearable sensors have been used to detect and differentiate between seizures, and in Parkinson’s disease (PD), sensors can be used to quantify motor symptoms and medication-induced side effects [13]. However, the clinical impact of wearable sensors remains uncertain. A systematic review of 56 studies on the use of wearable sensors in epilepsy, PD and stroke revealed that the evidence base is scarce for using wearable sensors in supporting medical decision-making [13]. In some other neurological disorders, such as Duchenne Muscular Dystrophy (DMD), wearable motion sensors have led to the development of a KineDMD ethomic biomarker that can assist in the prediction of disease progression in patients [16]. The stride velocity 95th centile (SV95C), based on magneto-inertial sensors, was the first digitally derived metric approved by the European Medicines Agency in 2023 [17] and heralds a new era of novel digital endpoints that could be used in clinical trials of movement disorders. Due to the rapid advancement of wearable devices for symptom tracking of various neurological disorders, it is important to evaluate the utility of physiological measures in monitoring disease states. Recently, a review has explored the application of wearable sensors in paediatric neurological conditions [18]. The review concluded that while wearable sensors have the potential to transform the management of paediatric neurology, more research will be needed to assess their clinical effectiveness.

### 1.2. Validity

Wearable sensors that capture movement using inertial measurement units (IMUs) have been investigated in studies of gait analysis. One study showed that validation metrics of IMUs had good to moderate correlation with optical motion capture for movement measures [19]. Despite this agreement, the authors also pointed out that study limitations highlight the uncertainty in validating IMUs. While wearables can help to monitor heart rate variability (HRV) and sleep in hospital settings, the lack of validation in hospital settings limits their accuracy as digital biomarkers [20]. Others have highlighted challenges related to data quality, generalisability and interpretability regarding automatic assessment of atypical motor development [21]. Sensors used for continuous monitoring of motor movements in infants require further validation before they can be widely used to detect changes in atypical motor development [21].

### 1.3. Feasibility

In infants, data have shown that out-of-hospital assessments for predicting motor ability are feasible using a multi-sensor wearable when combined with a deep learning algorithm [22]. This finding is significant as it offers an objective method for investigating early signs of developmental delay in children and demonstrates the feasibility of using wearable sensors to monitor movement-related development trajectories in vulnerable populations. The study supports a potential use of a similar approach in RTT where frequent hospital visits for routine clinical monitoring may not be practical.

### 1.4. Study Aim

The use of wearable sensors has gained momentum in the RTT population [9,18]; therefore, it would be vital to examine the clinical effectiveness of their use in this population. While some have highlighted the feasibility of their use in RTT [18], no study has systematically appraised the extant literature on wearable sensor use in individuals with RTT and assessed the quality of articles. The purpose of this systematic review was to answer the research question of whether wearable sensors are informative in monitoring disease states in RTT. To achieve this, the study had the following objectives:

(I) To perform a systematic review and quality appraisal on studies of wearable sensors in individuals with RTT;
(II) To determine whether studies on wearable sensor use in RTT can reveal clinically meaningful insights into disease states;
(III) To obtain a better understanding of the relationship between relevant physiological markers and disease states in RTT.

## 2. Methods

### 2.1. Search Strategy

This systematic review followed the guidance described in the Preferred Reporting Items for Systematic Reviews and Meta-Analyses (PRISMA) [23]. It was conducted using the following electronic databases: PubMed, PsycINFO, Embase and Web of Science. No date restriction was applied, and the truncation symbol (*) was used to capture as many records as possible. Jatinder Singh performed the search on 6 July 2025, and another search was conducted by A.M. and G.W. on 24 July 2025, in an independent and blinded manner. To assist with their searching, G.W. and A.M. exported the studies to Rayyan [24] to screen the studies and remove irrelevant literature. Rayyan is a widely used electronic platform for completing systematic literature reviews [24]. It enables automated blinding of researchers and incorporates user-friendly features for navigating the inclusion/exclusion process, reducing the risk for errors. A.M. and G.W. utilised the platform to screen articles identified via the search. Generic tokens like “Rett” were managed by screening the articles and excluding those that were not relevant to the field of RTT. To make the search as comprehensive as possible, a snowballing approach was also used [25] to identify any articles by searching the reference section of relevant article(s). No inter-rater agreement statistics were performed. However, following the search, Jatinder Singh, G.W. and A.M. discussed the articles for eligibility. Jatinder Singh, G.W. and A.M. resolved any conflicts before a consensus was reached, and a final list of eligible articles was produced. The PRISMA 2020 checklist is presented in Supplementary Information File S1.

### 2.2. Search Terms

((Rett Syndrome*) OR (Rett) OR (RTT)) AND ((Wearable device*) OR (Wearable sensor*) OR (Wearable system*) OR (Smart device*) OR (Wireless device*) OR (Fitness tracker*) OR (wearable technology) OR (Smart Watch) OR (accelerometer)).

### 2.3. Population Characteristics

The database searches focused exclusively on individuals with RTT.

### 2.4. Intervention

Studies that included information on wearable sensors, wearable devices, wearable systems, smart devices, wireless devices, fitness trackers, wearable technology, smart watches or accelerometers.

### 2.5. Eligibility Criteria

Inclusion Criteria

➢ Records (full-text articles in peer-reviewed journals).
➢ Individuals with RTT.
➢ The study focused on wearable devices (or similar)

Exclusion Criteria

➢ Records not available in English.
➢ Studies done using animal models (or not deemed relevant).
➢ Single cases or cases with two individuals or fewer.
➢ The following types of literature were excluded: reviews (all types), meta-analyses, preprints, letters, conference proceedings, protocols and book chapters.

### 2.6. Extraction of Data

Data extraction was performed by Jatinder Singh and then reviewed by G.W. and A.M. As part of this process, A.M. and G.W. reviewed the extracted data from the 12 eligible articles. Any discrepancies in the data extraction were resolved following agreement between Jatinder Singh, A.M. and G.W. Dual independent data extraction was not performed. The extracted data included the sample size, ethnicity reporting, study design, sample characteristics, assessment methods used, and key findings from the eligible articles.

### 2.7. Quality Appraisal of Included Articles

The quality appraisal of articles was performed using the Joanna Briggs Institute (JBI) checklist for the critical appraisal of studies in systematic reviews [26]. This tool was selected because it has previously been used for the assessment of studies in systematic reviews on autonomic dysregulation, sudden death and quality of life in RTT [27,28]. Each study was evaluated against a checklist comprising 11 items. Each of the 11 items was assessed using a score (yes = 1, no = 0 or unclear = 0). When an item did not apply to the study being appraised, the item was marked as not applicable (N/A) and excluded from the denominator, i.e., if 2/11 items are marked as N/A, the total score was based on the nine remaining items. Total scores were expressed as percentages and categorised according to levels of bias. Higher scores indicate a more robust methodological quality. Studies scoring above 70% were deemed to have a low risk of bias, those scoring between 50% and 69% were deemed to have a moderate risk of bias and those scoring less than 50% had a high risk of bias [29]. The quality appraisal was performed by the first author (Jatinder Singh) and then reviewed by the second (G.W.) and third author (A.M.). Any disagreements were discussed between Jatinder Singh, G.W. and A.M., and a consensus was reached before the quality appraisal was finalised.

## 3. Results

### 3.1. Article Characteristics

**Figure 1:**
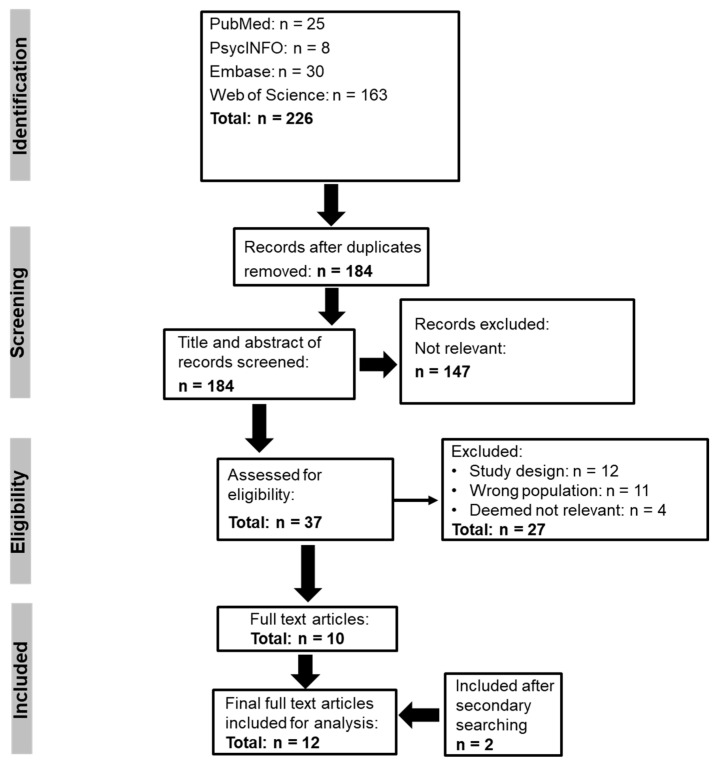
PRISMA flowchart.

Using the PRISMA guidelines, database searching identified 226 records, with 184 remaining after duplicates were removed. When screened against the eligibility criteria, 147 articles were deemed not relevant, with 37 articles remaining for full text screening. From the 37 articles, 12 were excluded due to study design, 11 due to population, and 4 were deemed not relevant, leaving 10 that met the eligibility criteria. Two additional articles were identified through ‘snowball’ searching, and in total, 12 articles were included in the final analyses (Figure 1). Four were pilot studies [30,31,32,33], four were observational studies [34,35,36,37], two were randomised clinical trials [38,39] and in another [40], the dataset was sourced from previous studies. One further study was also a retrospective analysis [41]. All studies either had individuals with a clinical diagnosis or a confirmed pathogenic variant of *MECP2*. The sample size ranged from 7 [31] to 47 study participants [41] with an age range of 1 year 8 months [33] to 41 years old [34]. Nine studies have utilised physiological monitoring with wearables in conjunction with subjective outcome measures [30,31,33,35,36,37,38,39,40]. The Empatica E4 device was used in 33% (4/12) of studies [31,32,34,35]. The remainder of the studies used different wearable devices, ranging from the YouCare Wearable Medical Device [30] to the sleep actigraph (Philips Actiwatch 2) [33], the ActivPAL uniaxial accelerometer and Stepwatch Activity Monitor (SAM) [36,37,39], Biostamp^®^ nPoint (a body-worn device) [40], and the LifeShirt wearable physiology measurement system [41]. In another study, activity, sleep and cardiorespiratory function were measured using the ActiGraph wGT3XBT and Hexoskin Smart Kit [38]. In three studies, machine learning approaches were used to investigate (I) sleep prediction that was able to differentiate between awake, non-rapid eye movement (REM) and REM sleep in RTT individuals [31], (II) use autonomic metrics to distinguish between females with RTT and females with Autism Spectrum Disorder (ASD) [32] and (III) classify low clinical severity RTT individuals from high clinical severity RTT individuals [40]. The data also showed that only 3 out of 12 (25%) studies reported on ethnicity [33,38,40]. The characteristics of each included article are presented in Table 1.

### 3.2. Study Heterogeneity

The study showed that there was significant heterogeneity in the wearable devices. It ranged from having different sensor modalities such as cardiac activity (electrocardiograms), electrodermal activity (EDA), photoplethysmography (PPG) and RIP (Respiratory Inductance Plethysmography), placement of devices (i.e., wrist or chest) and sampling rates. A range of devices were also used, including the ActivPAL, ActiGraph wGT3XBT, BioStamp^®^ nPoint, Empatica E4, Hexoskin, LifeShirt, Philips Actiwatch 2, Stepwatch Activity Monitor (SAM) and the YouCare Medical Device. The frequency of device usage varied. While the Empatica E4 device was used in four studies, this does not imply comparability of data outputs from the E4 devices used in the studies [31,32,34,35]. Therefore, no assumptions can be made about the weight of the physiological data captured. Measurements of EDA can be prone to conductance artefacts such as placement, sweat and ambient temperature. There were also differences in the studies using machine learning models. Class distribution varied, and different cross-validation strategies were used, such as the K-fold cross validation [31] and Leave-One-Out-Patient Cross-Validation (LOOCV) [40]. Data structure also varied. For sleep prediction, each model was trained on data consisting of 23 features [31]. In the study on RTT clinical severity, there were 18 ‘high severity’ visits for 10 patients and 14 ‘low severity’ visits for 11 patients. A total of 32 data points were used for model development [40]. Performance metrics were also not adequately explained in another study [32]. Given this heterogeneity across studies, no cross-study meta-analyses could be performed to examine between-device variability further.

### 3.3. Comparative Overview of Sensors

When further analyzing the different sensors used in the studies, their examination revealed some critical insights. Sensors can be broadly categorized into two areas. These are (I) sensors integrated into textiles and (II) wrist or patch-based sensors. Their use was also dependent on whether the sensor was used for home monitoring [30,41], sleep [31,33], assessment of autonomic dysregulation [34,35], physical activity [36,37,39] or evaluation of clinical severity [40]. The YouCare Smart T-shirt is a certified medical-grade device that integrates non-invasive sensors directly into washable fabric. The sensors can capture heart rate, respiratory rate, skin temperature and HRV and highlight the use of polymeric sensors on clothing using ink-jet printing [30]. More broadly, it supports the premise that textile garments equipped with wearable sensors can be used for continuous home monitoring of physiological signals in individuals with RTT. The Empatica E4 device features different sensors such as a PPG sensor, an EDA sensor, a 3-axis accelerometer, and an infrared thermopile. This device enables multi-modal data acquisition focused on autonomic and emotional dysregulation in RTT. In particular, the EDA measurement can provide valuable insights into tonic and phasic components, enabling different aspects of emotional and psychological responses in response to stress to be evaluated.

When looking more broadly across the use of sensors in RTT, other sensors such as ActiGraph xGT3XBT, Hekoskin, Biostamp^®^ nPoint, ActivePAL and SAM diversify the ecosystem regarding the activity, posture, sleep or ECG measurements with different placements, sampling rates and duration reported. For example, the Biostamp^®^ nPoint captures ECG and three-axis accelerometer data in four locations in the body. However, these four locations were not used for all study participants [40]. Sampling rates for ECG records were captured between 125 Hz and 250 Hz, and for three-axis acceleration sampling rates were 31.25 Hz and 62.5 Hz [40]. For the E4, sampling rates for the PPG sensor were 64 Hz, and for the accelerometer was 32 Hz [31,32]. For other devices such as the ActivePAL, sampling rates for the uniaxial accelerometer and inclinometer sensors were not specified [36,37,39]. Moreover, although sampling information for the YouCare Smart T-shirt was not mentioned, HRV was calculated from RR intervals using the (root mean square of successive RR interval differences [RMSSD]) using 10 S epochs [30]. Some studies were also able to identify artefacts in the raw data using previously reported algorithms [30] or for artefact removal in the BVP signal [31,34,35]. When viewed together, these findings demonstrate a growing portfolio of wearables in RTT, allowing different insights into the complex symptom landscape of RTT to be revealed. It also stresses the importance of methods of data analysis unique to this specific patient group. Common features in RTT are stereotypies and breath-holding episodes [1,2]. These symptoms may lead to signal interruptions and distortions and further emphasize the important role of artefact detection when analysing HRV and EDA in this population.

### 3.4. Quality Appraisal of Articles

The quality appraisal of the 12 studies is shown in Table 2. Some of the studies were exploratory studies [30,31,32] and therefore formal power calculations were not applicable. In other studies, no power calculation was provided [34,35], while some considered the sample size was insufficient for certain statistical comparisons [33,41] and this was recognised by other authors [37]. Those that used machine learning methods also acknowledged the small sample size [31] or for the development of the machine learning model [40]. Other studies had sufficient statistical power to detect a change. In one study, each ketamine dose level had 80% power [38] while another had a post hoc power of 0.78 [39]. Most studies considered relevant confounding factors when using wearables, such as device placement and individual variation [31]. Five (5) studies [30,32,34,35,38] scored ‘yes’ on item 11 on the JBI checklist for conflict of interest from industry or commercial sponsorship. The findings from the JBI checklist showed that all 12 studies had a low risk of bias, i.e., scoring above 70% demonstrating that these studies were of a high methodological quality. None of the studies evaluated in the quality appraisal were rated as having moderate (scoring between 50% and 69%) or a high risk of bias (scoring below 50%). Overall, the quality appraisal demonstrates that the studies evaluated in this systematic review were generally of robust methodological rigour. There is no standardised objective approach for measuring physiological data in individuals with RTT. Nevertheless, the quality appraisal did show that the Empatica E4 device was the most frequently used (4/12) wearable device in studies of RTT and does enable comparison between studies where the E4 device was used.

### 3.5. Biomarkers of Disease States

From the perspective of studies using the Empatica E4 device, the analysed studies demonstrated that sleep patterns could be predicted in RTT individuals with an accuracy of 85.1% [31], and high electrodermal activity (EDA) was associated with physical health deterioration [35]. In this context, differentiation of dysfunctional sleep patterns and monitoring of EDA can help in the monitoring of disease symptoms in RTT. While polysomnography (PSG) is the gold standard for monitoring sleep patterns [42], the data suggest that the Empatica device could provide a cost-effective and practical alternative for monitoring sleep disturbances in RTT. While there is no concordance on the use of wearable biosensors in RTT individuals, elevated EDA levels in RTT [35] can be coupled with the findings from the YouCare Wearable Medical Device, which showed that the percentage of maximum heart rate (HRmax%) and the heart rate to HRV Low-Frequency power (HR/LF) ratio are objective markers of fatigue, severity and different disease modalities such clinical sleep disorder and subclinical hypoxia in RTT [30]. In RTT patients, sleep dysfunction was positively correlated with normalized low-frequency power (LFnu) and total power. In contrast, subclinical hypoxia was positively correlated with the LF/HF ratio and total power [30]. These results reflect a close association with the level of autonomic nervous system activity, and when taken together, suggest that an overall change in the HRV spectrum with increased EDA can provide additional insights into the disease. In other studies, accelerometers such as the ActivPAL and the Stepwatch Activity Monitor (SAM) can help in assessing walking activities in RTT individuals [36,37,39]. This may help to monitor the impact of disease state and physical activity in individuals with RTT.

While it has been demonstrated in cases that Propranolol can reduce autonomic stress in RTT [30,43], it would be prudent to explore whether medication could be targeted to those RTT individuals who are more clinically vulnerable. Using the BioStamp^®^ nPoint biosensor, one of the analysed studies developed a machine learning model to assess clinical severity in RTT [40]. Importantly, this study showed that capturing the mean Deceleration Capacity (DC) of HR measured using the BioStamp^®^ nPoint sensor between 10 p.m. and 10 a.m. was the most popular feature for the development of the machine learning model. This finding lends support to the premise that, in RTT, night-time data may be the most suitable option for enhancing model performance when training a machine learning model to evaluate clinical severity in RTT.

## 4. Discussion

To the best of our knowledge, this is the first systematic review of wearable sensor studies in RTT, conducted according to the PRISMA criteria, and a critical appraisal of the eligible articles. When viewed across all studies, the findings from the wearables indicate that (I) high EDA, (II) HRmax%, and (III) HR/LF ratio are informative markers for monitoring disease states in RTT. Moreover, when combined with machine learning methods, wearables can (I) predict different sleep patterns and (II) classify individuals into low and high-severity categories. In our data analysis, the mean HR DC during 10 p.m. and 10 a.m., as measured using the BioStamp^®^ nPoint sensor, was the most popular feature for model development to predict RTT severity [40]. The DC of HR is a marker of vagal activity [44], and also encompasses the respiratory and sympathetic response [45]. It has been identified as a prognostic biomarker for high-risk individuals following myocardial infarction [46] but also a significant risk factor for other comorbidities [47], some of which are relevant for RTT, such as obstructive sleep apnoea [48] and antipsychotic-induced side effects [49].

While machine learning methods can be used to enhance our knowledge on physiological biomarkers in RTT, the findings from the studies should be viewed from the perspective of performance framing to avoid optimisation bias. The stratified K-fold cross-validation method is useful when there are imbalanced classes [50]. This method was used to evaluate individual models for sleep classification in RTT [31]. A potential limitation in this method is that stratified K-fold cross-validation may not include representative samples in the minority class. However, to overcome this, a Borderline-Synthetic Minority Oversampling Technique (SMOTE) was used on the minority class samples to balance classes, thereby strengthening model learning near decision boundaries [31]. Another study used the LOOCV approach when investigating RTT severity [40]. This model was chosen instead of the k-fold validation because using the LOOCV allowed more data points for training the model [40]. In LOOCV, the removed data point is used to test the model’s performance. This process may lead to high variability in the model’s performance, making it less reliable and potentially overfitting. The authors [40] suggested the creation of open-source repositories of datasets [51] to help mitigate this problem. Having a repository would allow for the training of better models but could also facilitate benchmarking of new machine learning models against the same data. To improve interoperability between repositories, the Findable, Accessible, Interoperable, and Reusable (FAIR) principles can be implemented so that data within repositories can be more usable [52]. This would be particularly valuable in RTT, where patient numbers are small and data scattered. Evidence has demonstrated that applying FAIR across eight databases containing *MECP2* genetic variants can improve understanding of the genotype-phenotype relationship in RTT [53]. In conclusion, by adopting Borderline-SMOTE and LOOCV to strengthen model learning, these techniques can improve the methodological rigour of studies, especially when dealing with imbalanced datasets and small samples. When applied correctly, they provide a fairer assessment of sleep and clinical severity prediction in RTT and minimise the risk of artificial performance framing.

In machine learning, predictor variables or features help to optimise training, and accurate feature selection reduces the likelihood of model over-fit [54]. Features also influence the performance of models. For example, evidence in ASD has shown that features derived from different predictor variables affect model performance [55,56]. In another study, extreme gradient boosting was the best-performing model for predicting ASD from family medical history [57]. As more studies in RTT emerge that adopt machine learning methods, it is unlikely that there will be a single standout machine learning model that outperforms all others, a premise also echoed by others [58]. In other diagnostic spheres, such as diabetes, the Shapley Additive Explanation (SHAP) method has been utilised to interpret the performance of machine learning models, thereby enhancing their clinical utility [59]. The SHAP method enables more accurate assessment of the contributing features in the model for prediction, thereby helping to identify which factors are most relevant to patient risk. In studies with small sample sizes, the SMOTE [60] has been employed to enhance model performance by increasing the number of cases. The SMOTE has been used in predicting the onset of ADHD in young people [61] and for increasing the number of synthetic ASD cases by five times in another study that utilised electronic health records and machine learning for predicting the risk of ASD in newborns [56]. When there are more majority class samples, there is a risk of misclassification of samples. In RTT, the Borderline-SMOTE was used to balance datasets [31]. This implies that variations in the SMOTE technique can be applied to machine learning models using data from RTT individuals to minimise the risk of misclassifying samples and improve the generalisability of findings. Future studies of wearable sensors that utilise machine learning approaches to predict risk factors in RTT could leverage the SHAP and SMOTE frameworks [31] to (I) assess the clinical utility of machine learning models and (II) increase the number of cases in training datasets. 

The study characteristics revealed that only 25% (3/12) of the studies [33,38,40] reported data on ethnicity. However, in these studies, many of the study participants were either Caucasian [33] or were white [38]. Ethnicity reporting in studies remains a significant challenge [62], and from the perspective of using biosensors it is an important factor to consider. There is evidence to suggest that pulse oximeters have increased error rates in those with darker skin tones [63]. However, it is unclear to what extent diverse skin types affect the accuracy of PPG sensors. Previous evidence has shown that devices with a PPG sensor are less effective at detecting light reflections in darker skin [64] and considered by others [18]. Another study that systematically validated wearables across a diverse range of skin tones showed no statistically significant difference in the accuracy of PPG data when assessed against different skin tones [65]. This has generated a meaningful debate in the field, highlighting issues regarding sample sizes and appropriateness of outcome measures [66,67]. It is unclear whether light penetration depths can be influenced by factors such as hair, sweat and ambient temperature [66,67]. Given the nature of how the PPG signal traverses the vasculature, skin epidermal thickness can lead to fluctuations in the PPG signal [68]. Currently, there is no data on PPG signals and skin tones in the RTT population. Only 25% of studies reported on participants’ ethnicity, and none examined how sensor accuracy varies with skin tones. Therefore, based on the findings of this systematic review, we cannot determine whether the findings from studies using wearable sensors apply to RTT individuals from diverse backgrounds. This highlights a significant gap in wearable sensor research. As the field is gaining momentum, it would be prudent to include diverse populations in wearable sensor research to minimise existing healthcare disparities.

Wearable sensors such as the Empatica wristband devices have been used to detect seizures and assess seizure-related autonomic dysregulation in individuals [69]. Using EDA and an algorithm to detect patterns associated with generalised tonic–clonic seizures (GTCS), in 2018, the Empatica Embrace was the first non-electroencephalogram (EEG) device to receive Food and Drug Administration (FDA) approval for use in adults and children > 6 years of age [70]. Evidence also suggests that EDA can increase during the pre-ictal stage in a small subset of individuals [71]. Another study has shown that the magnitude of PPG signals was higher during the pre-ictal and post-ictal period [72]. Children are noted to have greater EDA increases during the post-ictal period than adults [73]. The Empatica E4 device, in conjunction with machine learning approaches, has also been used to facilitate seizure detection (reviewed in González Barral and Servais [18]). When viewed together, robust objective data indicates that non-invasive wearable sensors can aid in seizure detection. We have previously surmised that pro-active surveillance of the autonomic profile in individuals with RTT could help manage epilepsy and lower the risk of Sudden Unexpected Death in Epilepsy (SUDEP) [74]. When using the Empatica device, the precursors of SUDEP, i.e., post-ictal generalised EEG suppression (PGES), may manifest as an unusually high EDA surge [69,75]. This finding is significant given the correlation between PGES >50 s and an elevated risk of SUDEP [76]. In a meta-analysis of EDA responses in 82/100 seizures, evidence suggests that there tends to be higher and longer EDA signals with GTCS and focal to bilateral tonic–clonic seizures (FBTCS) when compared to focal seizures (without FBTCS) [77]. Despite these findings, there is no consensus on a definition of an EDA response, either in terms of amplitude or length [77]. While we have previously indicated that sensors could potentially be used to monitor the risk of SUDEP in individuals with RTT [74], the data from the systematic review revealed that most of the studies did not investigate seizure activity in this population. In one study, HRV parameters were associated with EEG findings; however, the relationship between epileptiform activity and HRV parameters was unclear [30]. This finding highlights a significant knowledge gap in research exploring how wearable sensors can detect seizures or assess SUDEP risk in individuals with RTT. Seizures are frequent in RTT, and misdiagnosis (both overdiagnosis and underdiagnosis) is common. Prolonged EEG monitoring is challenging in the RTT population. This further highlights the importance of potential wearable sensor data in supporting timely and accurate diagnosis, as well as monitoring the response to treatment.

Disorders of gastrointestinal (GI) motility, such as gastroesophageal reflux disease (GERD) and constipation, are frequent in individuals with RTT [78]. A recent systematic review has indicated that GERD and other gastrointestinal (GI) disorders show abnormalities in HRV parameters, namely depressed vagal tone and sympathetic dominance [79]. Some noteworthy findings in GERD are a significant increase in the sympathovagal (LF/HF) ratio [80,81], while an increased sympathetic tone was noted for chronic refractory constipation [82] and sleep deficiency and overall autonomic dysfunction in individuals with constipation [83]. Individuals with RTT may be more prone to infections, particularly respiratory infections [84,85] and may also experience immune system dysfunction [86]. Recent evidence has demonstrated that multimodal biosensors utilising machine learning models can predict a systematic inflammatory response following a low-grade challenge with the influenza virus in healthy adults [87]. This finding has the potential to decrease the detection time of inflammation, even when symptoms are not immediately apparent and theoretically could have practical implications for individuals with RTT who are hypothesised to have chronic inflammation [88]. While wearable sensors have the potential to assist in the management of seizures, disorders of GI motility, and detect signs of chronic inflammation, the feasibility of their use to address these target areas in RTT is unknown, underscoring the importance of focusing research on these areas.

## 5. Limitations

The findings of this review should not be taken as confirmatory, but rather as associative. While we have suggested that SHAP and SMOTE frameworks could be helpful, the paucity of machine learning models using wearable sensors in RTT precludes meaningful insights into how effective these frameworks would be when developing models. Although the review extensively appraised the literature, we are aware that not all the relevant literature may have been covered. Furthermore, no formal inter-rater statistics were performed during the full-text screening phase. To mitigate this, two authors independently reviewed the literature, and a third author also reviewed the studies. Any conflicts regarding the eligibility of articles were resolved between the three authors before a consensus was reached. Dual independent data extraction was not performed, and this could introduce bias into the review process. However, to minimise this bias, data extraction was checked by Jatinder Singh, A.M. and G.W., and any discrepancies were resolved before consensus was reached. The systematic review was also not registered; however, it did adhere to the PRISMA checklist guidelines (File S1). We are also cautious in making an association regarding the accuracy of PPG sensors to individuals with diverse skin tones. While ethnicity reporting and health inequities in participation pose challenges to address, the evidence base suggests that further research is needed to determine whether the accuracy of PPG sensors is correlated with individuals of different skin tones. Finally, some of the sensors (E4 device, ActivPAL and SAM) were used in studies from the same research groups, which could increase bias, particularly with overlapping study samples. However, the studies met the PRISMA eligibility criteria as determined by three authors (Jatinder Singh, G.W. and A.M.) and were therefore included allowing them to be reviewed critically alongside other studies in the field.

## 6. Conclusions

**Figure 2:**
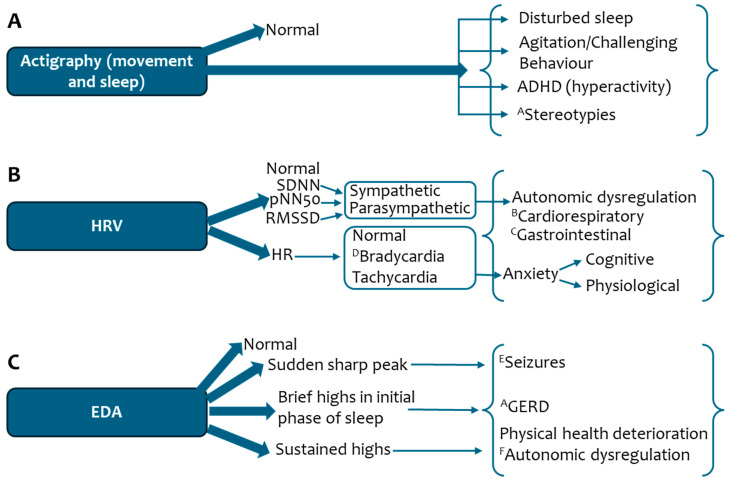
Physiological markers (Panel **A**: Actigraphy, Panel **B**: HRV and Panel **C**: EDA) and their relevance to pathologies in Rett syndrome. Abbreviations: ADHD (Attention Deficit Hyperactivity Disorder); ANS (Autonomic Nervous System); BMI (Body Mass Index); ECG (Electrocardiogram); EDA (Electrodermal Activity); FBTCS (Focal to Bilateral Tonic–Clonic Seizures); GERD (Gastroesophageal Reflux Disease); GTCS (Generalized Tonic–Clonic Seizures); HR (Heart Rate); HRV (Heart rate Variability); pNN50 (percentage of successive R-R intervals that differ by more than 50 ms); PEG (Percutaneous Endoscopic Gastrostomy); PGES (Post-ictal generalized EEG suppression); PPIs (Proton Pump Inhibitors); PRN (pro re nata); RMSSD (root mean square of successive differences); RTT (Rett syndrome); SDNN (standard deviation of all NN intervals); SSRI (Selective Serotonin Reuptake Inhibitors). ^A^ Preliminary finding and more data are required. ^B^ Cardiorespiratory is more related to breathing dysregulation. Kubios HRV software [90] can give respiratory rate estimation and provide information on breathing dysregulation. ^C^ More related to the parasympathetic component. ^D^ Can help to rule out a medical cause of bradycardia, including medication—avoid beta blockers. ^E^ PGES may present as an unusually high EDA surge. ^F^ Autonomic dysregulation is also prominent in seizures and GERD.

In conclusion, this systematic review extensively analysed studies of wearable sensors in RTT. It showed that high EDA, HRmax% and HR/LF ratios are informative for monitoring disease in RTT. Two studies also employed machine learning techniques for detecting sleep and assessing clinical severity. The SHAP and SMOTE frameworks can be utilised to advance future studies in RTT, specifically addressing the performance of machine learning models and small sample sizes. The lack of ethnicity reporting in studies highlights health inequities in participation and reporting in wearable sensor research in RTT. Limitations in symptom areas such as epilepsy and disorders of GI motility highlight a significant gap in the knowledge base. Given that the Empatica device was the most frequently used in the identified studies, to bridge this gap, different pathologies and their association with physiological markers (actigraphy, HRV and EDA) in RTT have been summarised (Figure 2). These clinical notes offer a valuable contextual insight into the interpretation of physiological markers captured using wearable sensors in individuals with RTT. Figure 2 describes how autonomic (HRV and EDA) and behavioural symptoms (disturbed sleep, agitation, hyperactivity and stereotypies) may be associated with measurable physiological signals. While further evidence is needed for the clinical utility of using wearable sensors in RTT, by linking physiological signals observed with symptoms in RTT, Figure 2 bridges the gap between sensor data and its clinical relevance, serving as a key resource of information for health professionals, and may help to facilitate the monitoring of disease symptoms in real-world settings. The research landscape is evolving with the emergence of new studies utilising wearable sensors. The 2025 Validation of Innovative Biosensors for Rett Autonomic Symptom Tracking (VIBRANT) study will use FDA-approved wearable biosensors to track symptoms, including HR, breathing, sleep, oxygen saturation and movement in individuals with RTT for up to 9 weeks [89]. The main goal of the VIBRANT study is to (I) evaluate the feasibility and reliability of collecting these physiological measurements in RTT and (II) compare the biosensor data to data collected during an overnight sleep study. In summary, while this review has highlighted challenges, wearable sensors are a burgeoning field with the potential to transform symptom management in RTT. Moving forward, their use, alongside traditional subjective outcome measures, will also be valuable for improving the efficacy of primary outcome measures in clinical trials in RTT.

**Notes:**

**(A)**  **Actigraphy**

Actigraphy helps to detect sleep patterns by recording movement during sleep.

I. Disturbed sleep: In RTT, the management of disturbed sleep depends on certain factors, such as difficulty falling asleep, frequent nighttime waking, or waking up too early in the morning. It also depends on the frequency of nights with disturbed sleep, which determines whether the patient requires a PRN or regular medication. Behavioural intervention and good sleep hygiene are the initial steps in the management of sleep disturbance, but sometimes patients might also need pharmacological intervention. The most commonly used medication for managing disturbed sleep is Melatonin, especially when falling asleep is a major issue. Nightmares have sometimes been linked to Melatonin use; however, these are uncommon. Other treatment options may include Clonidine, an antihypertensive with sedative properties that can also help in the management of ADHD and dystonia. Clonidine use requires close monitoring of blood pressure alongside monitoring of other side effects such as for patients who are already on a medication that can affect blood pressure. In cases where sleep disturbances are less frequent, Promethazine PRN could be considered as an alternative. Benzodiazepines are usually avoided due to their side effects of respiratory depression. In adult patients who have Generalized Anxiety Disorder, Mirtazapine could be a better alternative for individuals when management of both anxiety symptoms and disturbed sleep is required.
II. Agitation/Challenging behaviours could result in self-injury. This may be seen as increased, sudden motor movements along with increased HR. In the younger age group, non-pharmacological interventions are considered the first line of therapy. Where medication is used, antipsychotics may be prescribed in low doses. Aripiprazole in small dosages has been shown to improve challenging behaviour in RTT. Risperidone is another alternative which has been shown to help with challenging behaviour, especially in emergencies where Risperidone Quicklets could be used. Antipsychotics should be used sparingly in patients with higher BMI. They may also increase extrapyramidal symptoms such as increased muscle tone and excessive salivation. Promethazine can also be used as a PRN medication to help manage challenging behaviour in emergency use and where antipsychotics are contraindicated. If the underlying cause of challenging behaviour is related to anxiety, then targeting anxiety symptoms using anxiolytics or antidepressants could be more helpful.
III. Hyperactivity: The diagnosis of ADHD in patients with RTT could be challenging due to their limited mobility and stereotypies. In some cases, hyperactivity alongside other diagnostic features of ADHD can be present in patients. Hyperactivity may be managed using typical stimulants or non-stimulant ADHD medications. Clonidine and Guanfacine have sometimes been used for managing agitation/challenging behaviour in some patients if the initial strategies fail.
IV. Stereotypies: In less mobile patients, stereotypies (such as hand wringing and hand mouthing) may be exacerbated and present as frequent abnormal movement patterns on actigraphy. As a consequence, these patients may need further evaluation for injuries, chronic inflammation and callosities on the hands and around the mouth. Initially, these patients would benefit from non-pharmacological interventions to prevent further damage to the skin and associated areas.

**(B)**  **HRV:**

HRV is the variation in time between individual heartbeats and can be measured using HRV metrics.

I. Changes in the sympathetic metric (SDNN) and those responsible for vagally mediated HRV (RMSSD and pNN50) can provide information on autonomic dysregulation. There is preliminary evidence to suggest that Buspirone may be useful for managing the cardiorespiratory component of autonomic dysregulation, e.g., breathing dysrhythmias. However, in some patients, Buspirone can lead to worsening of constipation and be associated with discomfort and pain.
II. An increase in HR could manifest as a physiological response to anxiety or could be related to pain. In RTT, it can also be due to episodes of breath-holding. Anxiety can be associated with increased EDA. The cognitive component of anxiety can be managed using Sertraline, Buspirone, Fluoxetine or Fluvoxamine. Based on clinical experience with our patient group, Sertraline seems to have better outcomes where breathing dysregulation is minimal. In instances when breathing dysregulation is the key symptom that needs to be controlled alongside anxiety, Buspirone seems to be a better option. Given the immunomodulatory properties of Fluvoxamine [91], it may be an option to be considered in cases where patients have recurrent infections. However, regular monitoring of the ECG is warranted for QT prolongation.
III. The physiological component of anxiety can be managed using beta blockers such as Propranolol with close monitoring of blood pressure during the titration period. It should be avoided in patients with a history of bronchial asthma or bradycardia. Propranolol should be used with caution in patients who are already on medications that can decrease blood pressure.

**(C)**  **EDA:**

EDA is defined as changes in skin conductance and measures the sympathetic component of the ANS.

I. A sudden, longer-lasting surge may be associated with seizures, particularly GTCS and FBTCS [77]. Seizures can be managed with antiepileptics which a Neurologist prescribes.
II. Brief elevations in EDA due to discomfort arising from a postural change when lying down may be signs of GERD. However, further information is needed to substantiate this. Symptoms of GERD can be managed conservatively using common PPIs such as Pantoprazole, Lansoprazole or Esomeprazole. The formulation should be considered when choosing PPIs, depending on the mode of administration either orally or via the PEG.
III. Sustained and abnormally high EDA may reflect physical health deterioration with autonomic dysregulation (sympathetic dominance). Buspirone and Propranolol may help to manage autonomic dysregulation. Acute physical health problems such as infections and sepsis may need to be ruled out.

**(D)**  **EBAD:**

It is common to have abnormalities in actigraphy, HRV and EDA, which gives rise to emotional, behavioural, and autonomic dysregulation (EBAD) and is observed frequently in treatment non-responders [8].

## Figures and Tables

**Table 1:** Characteristics of Eligible Studies.

Source	*N* (RTT)	Study Design	Ethnicity Reported (Yes/No)	Sample Characteristics	Assessment Methods	Relevant Findings
[30]	10	Pilot monocentric study	No	Mean ± SD age of individuals was 18.3 ± 9.4 years (range: 4.7 to 35.5 years). Individuals had a pathogenic *MECP2* gene variant and had diagnostic criteria for typical RTT.	Heart rate (HR) variability parameters, respiratory rate and skin temperature measured using YouCare Wearable Medical Device. Indirect assessment of water vapour pressure and carbon dioxide. Clinical severity assessments included the CSS, MBA, RSBQ and MPSS.	The study showed that HRV parameters were elevated in paediatric patients. The HRV parameter (HRV HR/LF) ratio was associated with (I) phenotype severity, (II) disease progression, (III) clinical sleep disorder (IV), subclinical hypoxia and (V) EEG changes.
[31]	7	Analytical validation pilot study	No	Mean ± SD age of individuals was 7.22 ± 3.66 years (range: 4 to 16 years) and had a diagnosis of classical RTT confirmed genetically (*MECP2* mutation)	Physiological data (HRV, EDA, movement and temperature) was collected using the Empatica E4 device. Polysomnography (PSG) was done using the Vanderbilt Sleep Core Clinical assessment included the revised MBA. Machine learning models for sleep prediction.	Comparing to PSG, this study was able to predict three stages of sleep using machine learning models. The models were able to accurately (85.1%) differentiate between (I) awake, (II) non-rapid eye movement sleep and (III) rapid eye movement sleep.
[32]	10	Proof of concept exploratory study	No	Sample consisted of 10 female individuals with RTT (age range: 5–20 years) and 10 age and gender matched individuals with ASD (age range: 6–19 years) Individuals were confirmed as having RTT and diagnosis of ASD confirmed using a multimodal assessment.	Physiological parameters (HRV, movement and EDA) measured using Empatica E4 device.	Using machine learning methods, this study was able to use autonomic metrics to discriminate between RTT and ASD females with 95% accuracy.
[33]	13	Feasibility (pilot) study	Yes	Mean age: 9 years 5 months (range: 1 year 8 months to 17 years 1 month) with a confirmed *MECP2* mutation	Sleep actigraph (Philips Actiwatch 2) Parent reported sleep diary Child Sleep Habit’s Questionnaire	The sleep actigraph was useful in assessing sleep at home in RTT individuals.
[34]	45	Observational study	No	The study sample consisted of 45 subjects (44 female and 1 male) with a mean ± SD age 16.46 years ± 9.29 years Genetic mutation was known for 30 subjects, 13 subjects the genetic mutation was not known, and one subject had a clinical diagnosis.	HRV measures captured using the Empatica E4 device. Data was assessed during the day and night.	The key findings from the study showed HR decreased with age and is lower at night. The study concluded that sympathetic and parasympathetic measurements were higher during the day that night.
[35]	10	Observational study	No	Mean ± SD age of individuals was 11.87 ± 4.97 years (range: 6–20 years). Study participants had a clinical and genetic diagnosis of RTT	HRV and EDA parameters were measured using the Empatica E4 device. Clinical severity was assessed using the RTT anchored CGI.	The study showed that normalisation of EDA using Buspirone was associated with improvement of EBAD in individuals with RTT. Elevated EDA could be a biomarker for symptom deterioration in RTT.
[36]	26	Observational study	No	Median age [IQR]: 16.0 (9.4–20.6) years. All participants had a confirmed diagnosis of RTT (87% had a pathogenic *MECP2* variant)	ActivPAL accelerometer Modified Bouchard activity record	Study demonstrates the validation of the ActivPAL. Sedentary time in individuals with RTT can be captured using the ActivPAL.
[37]	26	Observational study	No	Mean age (SD): 18 years (8) and all participants either had a clinical or genetic diagnosis of RTT	Actigraph, ActivPAL and SAM Video recorded session of activities Gross motor scale for RTT	Three accelerometer devices assessed walking activity in individuals with RTT. The study that the SAM can allow the assessment of physical activity in individuals with RTT.
[38]	23	Placebo-controlled cross-overall RCT	Yes	Mean ± SD age of individuals in Ketamine-Placebo cohort: 8.4 ± 2.3 years Mean ± SD age of individuals in Placebo-Ketamine cohort: 7.7 ± 1.8 years All study participants had a confirmed pathogenic variant in *MECP2* gene Five-day treatment followed by a 9 day wash out before cross-over.	Activity was captured using the ActiGraph wGT3XBT and the Hexoskin Smart Kit detected activity, sleep and cardiorespiratory function. Clinical outcome measures.	The study demonstrated that Ketamine was safe and well-tolerated; however, no different in efficacy was observed between ketamine and placebo. Study findings also indicated that there were no changes in biosensor data were observed between ketamine and placebo.
[39]	38 ^Ψ^	Multicenter waitlist RCT	No	Study participants (one male) age: 6 years 11 months to 41 years 1 month and all had a genetically confirmed diagnosis of RTT. Patients were ambulatory but 47% required assistance.	ActivPAL (uniaxial accelerometer) for assessment of sedentary time and step count assessed using the SAM accelerometer at baseline, post-test and 12-week follow-up. Telehealth supported intervention. Clinical outcome measures.	In this study, sedentary time and daily steps were measured using the ActivPAL and SAM; however, no statistically significant differences were found post-test. The study concluded that telehealth intervention produced a minor improvement in physical activity.
[40]	20	Retrospective observational study Dataset was sourced from two studies ^¥^	Yes	Individuals were split into a low severity and high severity group. Median age for the low severity group was 8.5 years and for the high-severity group was 8 years. All participants were diagnosed with RTT.	Biostamp^®^ nPoint (body worn device). Assessment of CGI-S scores. Development of machine learning models to classify individuals with RTT based on low and high severity.	The study showed that a machine learning model using HRV and movement was able to classify low severity to high severity RTT individuals. A model utilizing HRV, MSTE and MSNR features resulted in the greatest AUC of 0.92.
[41]	47	Retrospective analysis	No	RTT individuals had a clinical diagnosis and pathogenic *MECP2* variant Age and gender matched controls (age range: 2–7 years).	HRV and RSA metrics were captured from ECG and RIP signals using the LifeShirt wearable physiology measurement system Assessment of cardiorespiratory coupling. Genotype-phenotype relationship.	When compared to controls, this study demonstrated that individuals with RTT had shifts towards sympathetic activation and/or parasympathetic inactivation. The study also showed that those RTT individuals with truncated pathogenic variants were the most different when compared to controls.

Notes: ^Ψ^ Number of participants included in the analysis. ^¥^ Data for this study was sourced from the Triheptanoin-clinical trial and the Outcome measures and biomarkers and development study. Abbreviations: ASD (Autism Spectrum Disorder); AUS (Area Under the Curve); CGI-S (Clinical Global Impression-Severity); CSS (Clinical Severity Score); EDA (Electrodermal Activity); EBAD (Emotional, Behavioural and Autonomic Dysregulation); EEG (electroencephalogram); HF (High Frequency); HRV (Heart Rate Variability); HR (Heart Rate); IQR (Interquartile Range); LF (Low Frequency); MBA (Motor Behavioural Assessment); MECP2 (gene that codes the methyl CpG binding protein 2); MPSS (Multi-System Profile of Symptoms Scale); MSNR (Multiscale Network Representation); MSTE (Multiscale Transfer Entropy); PSG (Polysomnography); RCT (Randomized Controlled Trial); RSBQ (Rett Syndrome Behaviour Questionnaire); RIP (Respiratory Inductance Plethysmography); RSA (Respiratory Sinus Arrhythmia) RTT (Rett syndrome); SAM (Stepwatch Activity Monitor); SD (Standard Deviation).

**Table 2:** Quality Assessment of Eligible Studies.

Study	Criteria											
	1. Was the Sample Characteristic of the Specific Population?	2. Were Patients Recruited in an Appropriate Way?	3. Was the Sample Size Sufficient to Power the Study?	4. Were the Study Participants Described in Detail and Fosters Comparison with Other Relevant Studies?	5. Was the Data Analysis Undertaken with Adequate Description of the Identified Sample?	6. Were Objective and Standard Criteria Used for the Measurements?	7. Were the Assessment and Measurement Methods Used Reliably?	8. Were the Statistical Analyses Used Appropriate?	9. Were Relevant Confounding Factors Described and Accounted for?	10. If Sub-Populations Were Identified, Were They Done According to Objective Criteria?	11. Was There a Conflict of Interest?	Total Score
[30]	Yes	Yes	N/A—the study was a pilot study.	Yes	Yes	Yes, both objective and standard assessment methods were used.	Yes	Yes, statistical tests were appropriate for different clinical domains and parameters evaluated.	Yes, methodological differences were described in the discussion.	N/A	Yes	9/9 (100%)
[31]	Yes	Yes	N/A as it was a pilot study. However, the authors acknowledge the small sample size of the study.	Yes, can be compared to other studies in which the Empatica E4 device was used.	Yes	Yes, objective measures included physiological monitoring and polysomnography. Standard assessment measures used was revised MBA.	Yes	Yes, feature selection and machine learning methods were described in detail.	Yes, placement of the E4 and variation between individuals was described.	N/A	No	8/10 (80%)
[32]	Yes	Yes	N/A (proof of concept study)	Yes, can be compared to other studies where the E4 device was used.	Yes	No—E4 device was used for capturing HRV parameters. No standard measurements of clinical assessments were used.	Yes	Yes	Unclear, the authors accounted for individuals with neurometabolic or neurodegenerative conditions, but no other information provided.	Yes, ASD subgroup was described.	Yes	8/10 (80%)
[33]	Yes	Yes	No and small sample size was recognised by authors	Yes, can be compared to previous work	Yes	Yes, sleep actigraph was used alongside sleep diary and sleep questionnaire.	Yes	Yes	Yes, limitations and lack of changes were due to reduced statistical power.	N/A	No	8/10 (80%)
[34]	Yes	Yes	No power calculation was provided	Yes, can be compared to studies were the Empatica E4 device was used.	Yes	No. The study used the E4 device to measure day and night HRV measurements but no standard criteria for clinical assessments were used.	Yes	Yes	Yes	N/A	Yes	8/10 (80%)
[35]	Yes	Yes	No	Yes, the Empatica E4 device was used.	Yes	Yes, physiological monitoring using the E4 and standard clinical assessment (RTT anchored CGI-I).	Yes	Yes	Yes, confounding factors were discussed.	Yes	Yes	10/11 (91%)
[36]	Yes	Yes	No and was acknowledged by the authors.	Yes—can be compared to other studies where the ActivPAL was used.	Yes	Yes	Yes	Yes	Yes. Limitations—due to small sample size and Bouchard activity record were mentioned.	N/A	No	8/10 (80%)
[37]	Yes	Yes	Although an adequate sample size was mentioned, no power calculation was provided.	Yes, where the SAM and was used.	Yes	Yes	Yes	Yes	Yes	N/A	No	8/10 (80%)
[38]	Yes	Yes	Yes—each dose level had 80% power	Yes	Yes	Yes, physiological measures and clinical outcome measures	Yes	Yes, and were based on primary safety and tolerability outcomes	Yes—dose limitations and methodological challenges were described.	N/A	Yes	10/10 (100%)
[39]	Yes	Yes	Yes—post hoc power was 0.78	Yes, to other studies where same accelerometers were used.	Yes	Yes—both physiological and clinical outcome measures were used.	Yes	Yes	Yes	N/A	No	9/10 (90%)
[40]	Yes	N/A—the data set was sourced from two other studies	Yes, the authors considered this aspect when developing the machine learning methods.	Unclear: Ages for low and high severity groups were provided but no genotype information. Machine learning methods for biomarkers needs to be validated in other studies.	Yes	Yes, both physiological and method for clinical severity was used.	Yes	Yes, the sample size was considered and factored into the study design, i.e., two groups (one mild and one severe).	Yes	N/A	No	7/9 (78%)
[41]	Yes	Yes	No—authors indicated that the sample size was not sufficient to power some comparisons.	Unclear—retrospective analysis from two previous studies. Study participants were not sufficiently described.	Yes	Yes, cardiorespiratory coupling was undertaken alongside genotype-phenotype study.	Yes	Yes	Yes—small sample size meant that the findings are of a suggestive nature	N/A	No	7/10 (70%)

Abbreviations: ASD (Autism Spectrum Disorder); CGI-I (Clinical Global Impression—Improvement); HRV (Heart Rate Variability); MBA (Motor Behavioural Assessment); N/A (not applicable); QoL (Quality of Life); SAM (StepWatch Activity Monitor); RTT (Rett Syndrome). Notes: (I) Ratings were defined as Yes (fully meeting the criterion = 1), No (not meeting the criterion = 0), Unclear (unclear to whether the criterion was met = 0) and N/A (criterion was not applicable, i.e., does not apply to the study being appraised. Items marked as N/A were excluded from the denominator). Total scores are presented as actual values and in percentages. (II) For item 11, studies were rated as ‘Yes’ if there was a conflict of interest from industry/commercial sponsorship. If a study reported no conflict of interest this was indicated by a ‘no’. When a conflict of interest statement could not be identified in the study this was rated as ‘unclear’. (III) Checklist items are described in Munn et al. (2014) [26].

## Data Availability

The data extracted and used in this systematic review was derived from information accessible from databases available in the public domain.

## Supplementary Materials

The following supporting information can be downloaded at: https://www.mdpi.com/article/10.3390/s25216697/s1, File S1: PRISMA checklist.
